# Mental and Behavioral Health Services Provided by Schools in the United States: A National Survey of School Nurses*

**DOI:** 10.1111/josh.70067

**Published:** 2025-08-15

**Authors:** Paige C. Chardavoyne, Neha Gupta, Marcus Erdman, Susan J. Boehmer, Robert P. Olympia

**Affiliations:** ^1^ Penn State Milton S. Hershey Medical Center Pennsylvania State University College of Medicine Hershey Pennsylvania USA; ^2^ Department of Psychiatry and Behavioral Medicine Medical College of Wisconsin Milwaukee Wisconsin USA; ^3^ Department of Emergency Medicine George Washington University Washington DC USA; ^4^ Department of Pediatrics, US Air Force—673 Medical Group Joint Base Elmendorf‐Richardson Anchorage Alaska USA; ^5^ Department of Public Health Services Penn State Milton S. Hershey Medical Center Hershey Pennsylvania USA; ^6^ Departments of Emergency Medicine and Pediatrics Penn State Hershey Medical Center Hershey Pennsylvania USA

**Keywords:** crisis intervention, emergency care, mental health, school nurse education, screening/risk identification

## Abstract

**Background:**

School nurses are often tasked with identifying and managing mental and behavioral health concerns. The objective of this study was to better understand school nurse and school preparedness to identify and manage mental health conditions and psychological stressors.

**Methods:**

The authors developed a questionnaire, which was electronically distributed to several National Association of School Nurses members during the 2021–2022 school year.

**Results:**

A total of 994 questionnaires were completed and analyzed, a 50% response rate. Of school nurse respondents, 76% felt responsible for identifying depression; 40% reported that their school has naloxone; 22% reported that their school has an emergency preparedness and response plan for opioid overdose; and 18% screened for bullying in the past year.

**Implications for School Health Policy, Practice, and Equity:**

School policies and guidelines for the critical areas identified in the present study should be considered.

**Conclusions:**

Based on our national sample of school nurses, we have identified strengths and areas for improvement in school nurse and school preparedness to identify and manage mental health conditions and psychological stressors.

## 1 Introduction

According to the Center for Disease Control and Prevention (CDC), in 2018–2019, approximately 13% of children aged 3–17 years had a diagnosed mental health condition [1]. Common mental health conditions in children and adolescents include anxiety, depression, attention‐deficit/hyperactivity disorder, and substance use disorders. The mental health of children and adolescents, particularly symptoms of depression and anxiety, worsened during the COVID‐19 pandemic [2]. A more recent estimate from the CDC suggests that up to 20% of children may experience a mental health condition annually [3]. Adverse childhood experiences (ACEs), such as caregiver abuse, intimate partner violence, and bullying, are psychological risk factors for developing a mental health condition during the lifespan. Early identification of ACEs and early intervention are paramount in attempting to mitigate future impact [4].

School nurses have been regarded as a connection between education and healthcare. According to the National Association of School Nurses (NASN), an organization whose mission is to optimize student health and learning by advancing the practice of school nursing, one of the many responsibilities of school nurses is “providing mental health support to students in need” [5]. This duty is further detailed in the NASN Position Statement titled “The Behavioral Health and Wellness of Students” originally published in 2022 [6]. In this statement, the NASN calls on school nurses to identify behavioral health concerns, highlighting psychological stressors, behavioral concerns, mood symptoms (anxiety and depression), and suicidality, and to assist with referral to appropriate community resources [6]. Given wait times with accessing community resources, school nurses and school staff may find themselves working with students in need as they bridge to necessary treatment. To address this access barrier, comprehensive school‐based mental and behavioral health services have aimed to incorporate emotional, behavioral, and social support into the existing school system. These services also involve mental health screening to identify students who may currently have or may be at risk for mental health concerns [7]. Benefits, including early detection of and treatment for mental health conditions, and challenges, including lack of awareness of screening practices and resource limitations, of this type of screening have been identified [8]. Yet, research suggests that regular mental health screening is taking place in a minority of schools, 20% or less [9].

With the mental health‐related demands placed on school nurses, the objective of this study was to better understand school nurse and school preparedness to identify and manage mental health conditions and psychological stressors. Topics included depression, suicidality, substance use, gender topics, child maltreatment, bullying, and relationship abuse. We hypothesized that school nurses would be involved in identifying mental health conditions and psychological stressors, but that areas for improvement with this and school processes would be identified.

## 2 Methods

### 2.1 Participants

The NASN is a nonprofit specialty nursing organization representing school nurses exclusively, which has 19,000 members and 50 affiliates, including the District of Columbia. Potential participants were 2000 members of the NASN. Per NASN research protocol, to limit the number of research requests that members receive from investigators, the questionnaire was sent electronically to 2000 members, the maximum number. Demographics of the 2000 randomly selected NASN members were not made available; therefore, responders and non‐responders could not be compared.

### 2.2 Instrumentation

The authors developed an electronic questionnaire which included: (1) school nurse demographics (professional licensure, highest level of education, and years practicing); (2) school demographics including setting (urban, suburban, and rural), region (Northeast, South, Midwest, and West), grades primarily responsible for (pre‐kindergarten and elementary, middle, and high school), and other school staff (guidance counselor, psychologist, social worker, and psychiatrist); and (3) protocols for identifying and managing common mental health conditions and psychological stressors, such as depression (3 questions), suicidality (3 questions), child maltreatment (2 questions), substance use and abuse (3 questions), bullying (2 questions), gender topics (1 question), and relationship abuse (2 questions). All questions were closed‐ended.

### 2.3 Procedure

This was a cross‐sectional observational study during the 2021–2022 academic school year. The NASN research coordinator was contacted in 2021 for assistance with questionnaire distribution. Per NASN research protocol, a NASN Membership Outreach Request Form for Research (requiring a biosketch of the senior author, abstract of the study, copy of the questionnaire, documentation of IRB approval, and timeline of questionnaire distribution) was submitted, reviewed, and approved. NASN administrative staff emailed a link to the study questionnaire to the same 2000 randomly selected NASN members on three separate occasions (November 2021, May 2022, and August 2022). A letter of informed consent was sent with the electronic questionnaire. After the first distribution, members were instructed not to complete the questionnaire if they had previously submitted it. No incentives were offered for participating in the study.

### 2.4 Data Analysis

Data was collected and organized using REDCap [10, 11]. A statistician (author SB) reported descriptive statistics for all response variables and calculated 95% confidence intervals by standard methods. The study was approved by the Institutional Review Board at the senior author's institution.

## 3 Results

A total of 994 questionnaires were completed and analyzed, a 50% response rate. The majority of respondents were registered nurses (98%) with the highest level of education reported as a Bachelor of Science in Nursing (53%) working in a suburban setting (48%) in the Northeastern and Southern United States (28%) (Table 1). The respondents' mean number of years working in school health was 12 years. Most respondents were responsible for pre‐kindergarten and elementary students (71%) with roughly half reporting being responsible for middle school (52%) and high school (46%) students. Respondents were not asked how many schools they covered; however, it appears that many respondents were responsible for more than one school.

**TABLE 1:** Demographics of respondents, reported as numerator/denominator, %, (95% CI) except for “Mean number of years as a school health provider” reported as mean, standard deviation, (95% CI).

		All responses
Professional licensure	Licensed Practical Nurse (LPN)/Licensed Vocational Nurse (LVN)	16/980, 1.6%, (1–2.6)
	Registered Nurse (RN)	947/980, 98.3%, (95.3–97.6)
	Advanced Registered Practice Nurse (ARPN)	15/980, 1.5%, (0.9–2.5)
	Other	2/980, 0.2%, (0.1–0.7)
Highest level of education	Associated Degree (AD)	127/978, 13%, (11–15.2)
	Bachelor of Science in Nursing (BSN)	513/978, 52.5%, (49.3–55.6)
	Masters	298/978, 30.5%, (27.7–33.4)
	Doctor of Nursing Practice (DNP)	4/978, 0.4%, (0.2–1.1)
	Doctor of Philosophy (PhD)	4/978, 0.4%, (0.2–1.1)
	Other	32/978, 3.3%, (2.3–4.6)
Mean number of years as a school health provider		12.3 ± 9.05 (11.7–12.8)
School setting	Urban	202/958, 21.1%, (18.6–23.8)
	Suburban	459/958, 47.9% (44.8–51.1)
	Rural	297/958, 31%, (28.2–34)
School region	Northeast	276/977, 28.3%, (25.5–31.2)
	South	274/977, 28.1%, (25.3–30.9)
	Midwest	226/977, 23.1%, (20.6–25.9)
	West	201/977, 20.6%, (18.2–23.2)
Grades primarily responsible for (was able to choose more than one response)	Pre‐kindergarten and elementary	704/994, 70.8%, (67.9–73.6)
	Middle school (6th–8th)	515/994, 51.8%, (48.7–54.9)
	High school	459/994, 46.2%, (43.1–49.3)
School staff includes the following:	Guidance counselor	722/958; 75.4% (72.5–78)
	Psychologist	483/958; 50.4% (47.3–53.6)
	Social worker	464/958; 48.4% (45.3–51.6)
	Psychiatrist	40/958; 4.2% (3.1–5.6)

In our sample, 75% of schools staffed a guidance counselor, 50% of schools staffed a psychologist, 4% staffed a psychiatrist, and 48% staffed a social worker (Table 1). Stratification by school setting and region revealed some statistically significant differences in staffing. Schools in rural areas more frequently staffed guidance counselors and less frequently staffed psychologists than schools in urban and suburban areas. Further, schools in the Southern United States less frequently staffed psychologists than schools in the Northeastern, Midwestern, and Western United States to a statistically significant degree.

Findings related to identifying and managing specific mental health conditions and psychological stressors are listed in Table 2. Regarding identification, of school nurse respondents, 76% felt responsible for identifying depression; 58% felt responsible for screening for child maltreatment; 52% received formal training in the recognition and management of adolescent relationship abuse (physical, sexual, or emotional); 18% screened for bullying in the past year; and 12% utilized screening, brief intervention, and referral to treatment (SBIRT) to address substance use.

**TABLE 2:** School‐based mental and behavioral health services, reported as numerator/denominator, %, (95% CI).

		All responses
Depression and suicidal thoughts		
School nurse is responsible for identifying students with depression		604/797; 75.8% (72.7–78.6)
School has guidelines for managing students with suicidal thoughts or actions		711/750; 94.8% (93–96.2)
School nurse has performed a risk assessment for self‐directed violence in the past year		231/801; 28.8% (25.8–32.1)
School has counseling and referral process in place for students affected by mass casualty incidents, death of student or staff member, or other grief or anxiety provoking events		524/958; 54.7% (51.5–57.8)
School nurse has provided emotional support for recovering students dealing with depression and other mental health disorders associated with concussions		374/958; 39% (36–42.2)
Child maltreatment		
School nurse is responsible for screening students for child maltreatment		476/816; 58.3% (54.9–61.7)
Has reported child maltreatment in the past year		477/825; 57.8% (54.4–61.1)
Gender topics		
School allows transgender and gender non‐binary students access to restrooms and locker rooms based on their gender identity and not matched to their genitalia		309/524; 59% (54.7–63.1)
Substance use and abuse		
School has an emergency preparedness and response plan for students with opioid related overdose		208/958; 21.7% (19.2–24.4)
School has the immediate availability of naloxone		382/958, 39.9%, (36.8–43)
School conducts random drug testing for students		72/781; 9.2% (7.4–11.5)
Screening method for substance use:	SBIRT (screening, brief intervention, and referral to treatment)	117/958; 12.2% (10.3–14.4)
	CRAFFT (car, relax, alone, forget, friends, trouble)	21/958; 2.2% (1.4–3.3)
	DAST‐A (drug abuse screening test for adolescents)	10/958; 1% (0.6–1.9)
	ASSIST (American stop smoking intervention study)	8/958; 0.8% (0.4–1.6)
	AUDIT (alcohol use disorder identification test)	7/958; 0.7% (0.4–1.5)
	CAGE‐AID (adapted to include drugs)	5/959; 0.5% (0.2–1.2)
	CAGE (cut down, annoyed, guilty, and eye opener)	4/958; 0.4% (0.2–1.1)
Bullying		
School has hosted education or training about bullying for the following:	Students	63/958; 6.6% (5.2–8.3)
	School staff	57/958; 5.9% (4.6–7.6)
	Bus drivers	11/957; 1.1% (0.6–2.1)
	Coaches	13/956; 1.4% (0.8–2.3)
School nurse has performed the following over the past year:	Screened for bullying during regular health assessments and/or sport physicals	172/958; 18% (15.7–20.5)
	Worked to develop action plans to combat bullying	90/958; 9.4% (7.7–11.4)
	Encouraged reporting of bullying behavior	493/958; 51.5% (48.3–54.6)
	Specifically asked students in high‐risk groups if they are experiencing bullying	247/958; 25.8% (23.1–28.7)
	Spoken with any at‐risk or identified “bullied” students	316/958; 33% (30.1–36)
	Asked staff for anti‐bullying ideas that can be implemented at your school	83/958; 8.7% (7–10.6)
	Asked parents for input/involvement regarding anti‐bullying efforts	69/958; 7.2% (5.7–9)
	Facilitated an anti‐bullying support club	12/962; 1.2% (0.7–2.2)
Relationship abuse		
School nurse has received formal training in the recognition and management of adolescent relationship abuse?		435/839; 51.8% (48.5–55.2)
School nurse is involved in the following:	Discussing the gravity of healthy relationships with students	177/958; 18.5% (16.2–21.1)
	Defining abuse in adolescent relationship for students	121/958; 12.6% (10.7–14.9)
	Conducting targeted assessments for adolescent relationship abuse	85/958; 8.9% (7.2–10.8)
	Intervening with a safe, student‐centered approach, including making referrals to local domestic violence and family planning/adolescent health partners	255/958; 26.6% (23.9–29.5)

Regarding management of school nurse respondents, 58% reported child maltreatment in the past year; 52% encouraged the reporting of bullying behavior; 39% provided emotional support for students dealing with mental health symptoms associated with concussions; 27% intervened with relationship abuse by making referrals to local domestic violence and family planning/adolescent health partners; 26% specifically asked students in high‐risk groups if they were experiencing bullying; 19% discussed the gravity of healthy relationships with students; 13% defined abuse in adolescent relationships for students; and 9% conducted targeted assessments for adolescent relationship abuse.

Within the schools where the respondents worked, 95% reported that their school has guidelines for managing students with suicidal thoughts or actions; 59% reported that their school allows transgender and gender nonbinary students access to restrooms and locker rooms based on gender identity; 55% reported that their school has a counseling and referral process in place for students affected by mass casualty or other grief provoking events; 40% reported that their school has naloxone immediately available; 22% reported that their school has an emergency preparedness and response plan for students with opioid related overdoses; and less than 10% reported that their school has hosted education or training about bullying for students and school staff.

## 4 Discussion

The objective of this study was to better understand school nurse and school preparedness to identify and manage mental health conditions and psychological stressors, including depression, suicidality, substance use, gender topics, child maltreatment, bullying, and relationship abuse. We hypothesized that school nurses would be involved in identifying mental health conditions and psychological stressors; however, areas for improvement with this and school processes would be identified. Findings from the present study suggest that, in particular, improvements in school nurse and school identification and management of substance use concerns and bullying warrant further attention.

The American Academy of Pediatrics and the Substance Abuse and Mental Health Services Administration (SAMHSA) recommend universal adolescent substance use screening, brief intervention, and referral to treatment (SBIRT) across settings where adolescents receive healthcare [12]. Despite this, only 12% of responding school nurses in the present study reported using substance use SBIRT. Though low, this is consistent with previous studies indicating rates of general mental health screening in schools below 20% [9]. A recent analysis of previous studies has highlighted some of the potential challenges with mental health screening in schools, such as lack of support for identified students, concerns with stigmatizing students, possible negative reactions from students' caregivers, and a lack of awareness of appropriate screening practices [8]. The latter is consistent with concerns that school nurses are unprepared to appropriately identify and manage students' mental health needs due to a lack of training [13]. To address training gaps in the area of student substance use identification specifically, the NASN has developed online learning modules to help those better understand assessment tools, such as the car, relax, alone, forget, friends, trouble (CRAFFT) [14].

Our findings also identified areas where school nurses and schools can improve when it comes to managing substance use. In the present study, only 22% of school nurse respondents reported that their school has an emergency preparedness and response plan for students with opioid related overdose and only 40% reported that their school has immediate access to naloxone. Since data collection, the NASN released a position statement stating that all individuals at all schools should have access to naloxone. They also called on school nurses to be involved in “planning, coordinating, and implementing evidence‐based emergency preparedness and response actions and essential healthcare for opioid overdose” [15]. With this new call to action, the authors anticipate that if the present study were repeated, there may be a higher percentage of school nurses reporting that their school has an emergency preparedness and response plan for students with opioid related overdose and has immediate access to naloxone. This is an area for future exploration.

The present study also highlighted some areas for improvement regarding bullying. In a 2021–2023 national survey of adolescents aged 12–17, 34% reported being bullied in the last year [16]. The NASN, in their 2018 position statement titled “Bullying and Cyberbullying ‐ Prevention in Schools,” acknowledged the consequences of bullying on developing children, encouraged school nurses to provide student‐centered care related to the topic, and suggested working with other school staff to facilitate bullying‐related interventions [17]. Our research identified areas for improvement in discussing bullying with students and in advocating for anti‐bullying efforts. Though not as frequent as desired, the most frequently reported area was encouraging reporting of bullying behavior (52%). The least frequently reported area involved directed anti‐bullying efforts, including developing action plans to combat bullying (9%). Addressing these areas, along with increasing bullying education and training for students, school staff, bus drivers, and coaches, which was present in the schools of a small minority of respondents, would be positive steps in managing the important issue.

Data from the present study suggests higher prevalence of identification and management at the school nurse and school level of depression and suicidality, child maltreatment, gender topics, and relationship abuse, though some areas for continued improvement remain. The 2022 NASN position statement titled “The Behavioral Health and Wellness of Students” highlights the unique role school nurses can have in identifying mental health conditions and directing students to additional care [6]. In our study, 76% of school nurse respondents felt responsible for identifying students with depression. Within mental health, topics of concern include suicidality and child maltreatment. A 2019 survey of high school students reported that 15.7% had made a suicide plan and 8.9% had attempted suicide [18].

Over 90% of respondents reported that their school has guidelines for managing students with suicidal thoughts or actions. Just over half (58%) of respondents indicated that they feel responsible for screening students for child maltreatment (physical/sexual/psychological abuse, neglect, or trafficking) and have reported child maltreatment in the past year. Given the unfortunate prevalence and downstream effects, screening for child maltreatment is paramount, as identification and intervention can have a large impact on trajectory [19]. Taken together, managing students with suicidal thoughts and actions appears to be a point of strength at the schools our respondents work at. However, there is room for improvement in screening for child maltreatment.

Transgender and gender minority youth have demonstrated improved health outcomes (mentally and physically) in supportive environments [20]. The NASN in a 2013 position statement titled “Sexual Orientation and Gender Identity/Expression (Sexual Minority Students): School Nurse Practice” highlighted the school setting as an opportunity to create a safe and supportive environment for students [21]. For transgender and non‐binary youth, areas of potential concern are bathrooms and locker rooms. Gender minority youth are at increased risk for sexual violence [22], but research supports that this risk in many gender minority groups is decreased when school restroom and locker room policies are less restrictive [23]. In our sample, 59% of school nurse respondents reported that their school allows transgender and gender nonbinary students access to restrooms and locker rooms based on gender identity rather than their genitalia. Research into additional means to support gender minority youth would identify other areas for continued progress.

Another important issue when working with school‐aged children is intimate partner violence, a topic which was addressed in 2010 by the American Academy of Pediatrics' clinical report titled “Intimate Partner Violence: The Role of the Pediatrician” [24]. Intimate partner violence is common and patterns can begin early with adolescents having a high rate of intimate partner violence [24]. Given this and the potential short‐ and long‐term physical and mental health effects [24], early interventions to identify intimate partner violence and provide appropriate resources are paramount; however, the issue needs to be handled carefully. Our research revealed that just over half of respondents (52%) had received formal training in the recognition and management of adolescent relationship abuse (physical, sexual, or emotional). However, few reported involvement in the following roles: intervening with referrals to appropriate resources (27%), discussing the gravity of healthy relationships (19%), defining abuse in adolescent relationships for students (13%), and conducting targeted assessments for adolescent relationship abuse (9%). Perhaps another healthcare worker in the school setting is involved in these roles; however, even if that is the case, it would likely be beneficial to have school nurses involved as well. Further research into this topic, including what barriers might exist for school nurse involvement in this area, would be of interest.

## 5 Implications for School Health Policy, Practice, and Equity

School‐aged children spend a significant proportion of their time in schools. This presents an opportunity to identify and manage mental health and psychosocial stressors in this setting. School nurses are well positioned to lead these efforts given their nursing education and integration into the school environment. In the present study, most school nurse respondents reported being involved with screening for depression and suicidality. This may reflect more school policies, guidelines, and school nurse education in these areas. Similar interventions should be extended to the identified critical areas of student substance use and bullying. Schools should aim to improve access to school‐based mental health interventions for all students.

## 6 Limitations

There are several limitations to the present study. First, our respondents were all members of the NASN who may be more aware of the NASN position statements and best‐practice guidelines. Thus, our data may represent practices of the NASN members versus school nurse nonmembers, which may limit generalizability. Second, through the NASN member respondents, we learned about guidelines and practices in the schools they worked. However, we were not able to gain information about procedures at schools that do not have a school nurse or whose school nurse is not a member of the NASN. Third, the questionnaire we used was not validated but instead developed by the authors to meet study objectives. Finally, the questionnaire was distributed in 2021 and 2022, which was during the COVID‐19 pandemic. It is possible that reported school nurse and school practices may have been impacted by this. Despite limitations, the authors had a 50% response rate among the NASN member nurses working in schools of various settings and regions in the United States.

## 7 Conclusions

In conclusion, the objective of this study was to better understand school nurse and school preparedness to identify and manage mental health conditions and psychological stressors, including depression, suicidality, substance use, gender topics, child maltreatment, bullying, and relationship abuse. Findings from the present study suggest that improvements in the identification and management of substance use concerns and bullying at the school nurse and school level in particular warrant further attention. However, opportunities for improvement in several of the other studied topic areas still remain. The authors have identified several limitations and areas for further exploration, such as the reassessment of school nurse and school preparedness for opioid overdose considering recent guidelines.

## Disclosure

IRB approval was obtained from the senior author's institution for this research study.

## Conflicts of Interest

The authors declare no conflicts of interest.

## Data Availability

The data that support the findings of this study are available from the corresponding author upon reasonable request.
